# Differential Expression of Long Noncoding RNAs in Murine Myoblasts After Short Hairpin RNA-Mediated Dysferlin Silencing In Vitro: Microarray Profiling

**DOI:** 10.2196/33186

**Published:** 2022-06-17

**Authors:** Richa Singhal, Rachel Lukose, Gwenyth Carr, Afsoon Moktar, Ana Lucia Gonzales-Urday, Eric C Rouchka, Bathri N Vajravelu

**Affiliations:** 1 Department of Biochemistry and Molecular Genetics KY IDeA Networks of Biomedical Research Excellence Bioinformatics Core University of Louisville Louisville, KY United States; 2 Department of Physician Assistant Studies Massachusetts College of Pharmacy and Health Sciences Boston, MA United States; 3 Department of Medical and Molecular Biology, School of Arts and Sciences Massachusetts College of Pharmacy and Health Sciences Boston, MA United States; 4 Department of Pharmaceutical Sciences Massachusetts College of Pharmacy and Health Sciences Boston, MA United States

**Keywords:** dysferlinopathy, long noncoding RNAs, lncRNA, abnormal expression, muscular dystrophy, limb-girdle muscular dystrophy 2B, LGMD-2B, messenger RNA, mRNA, quantitative real-time polymerase chain reaction, qRT-PCR, gene ontology, bioinformatics, transcription, noncoding RNA, protein expression

## Abstract

**Background:**

Long noncoding RNAs (lncRNAs) are noncoding RNA transcripts greater than 200 nucleotides in length and are known to play a role in regulating the transcription of genes involved in vital cellular functions. We hypothesized the disease process in dysferlinopathy is linked to an aberrant expression of lncRNAs and messenger RNAs (mRNAs).

**Objective:**

In this study, we compared the lncRNA and mRNA expression profiles between wild-type and dysferlin-deficient murine myoblasts (C2C12 cells).

**Methods:**

LncRNA and mRNA expression profiling were performed using a microarray. Several lncRNAs with differential expression were validated using quantitative real-time polymerase chain reaction. Gene Ontology (GO) analysis was performed to understand the functional role of the differentially expressed mRNAs. Further bioinformatics analysis was used to explore the potential function, lncRNA-mRNA correlation, and potential targets of the differentially expressed lncRNAs.

**Results:**

We found 3195 lncRNAs and 1966 mRNAs that were differentially expressed. The chromosomal distribution of the differentially expressed lncRNAs and mRNAs was unequal, with chromosome 2 having the highest number of lncRNAs and chromosome 7 having the highest number of mRNAs that were differentially expressed. Pathway analysis of the differentially expressed genes indicated the involvement of several signaling pathways including PI3K-Akt, Hippo, and pathways regulating the pluripotency of stem cells. The differentially expressed genes were also enriched for the GO terms, *developmental process* and *muscle system process*. Network analysis identified 8 statistically significant (*P*<.05) network objects from the upregulated lncRNAs and 3 statistically significant network objects from the downregulated lncRNAs.

**Conclusions:**

Our results thus far imply that dysferlinopathy is associated with an aberrant expression of multiple lncRNAs, many of which may have a specific function in the disease process. GO terms and network analysis suggest a muscle-specific role for these lncRNAs. To elucidate the specific roles of these abnormally expressed noncoding RNAs, further studies engineering their expression are required.

## Introduction

Dysferlinopathy is a type of muscular dystrophy, which are a group of inherited muscle degenerative disorders characterized by progressive muscle weakness. It is caused by the deficiency of dysferlin, a type II transmembrane protein that is highly expressed in skeletal muscle and the heart. Dysferlin interacts with several proteins by acting as a scaffold and plays a crucial role in calcium-dependent membrane repair in skeletal muscles [1]. Dysferlin deficiency in the muscles is characterized by vesicular accumulations, sarcolemmal disruptions, defective myogenesis, and increased inflammation [2], both in mouse models and in humans [3]. Dysferlinopathy has 2 subtypes—limb-girdle muscular dystrophy (LGMD) 2B and Miyoshi myopathy. LGMD-2B involves the proximal muscles of the limb and trunk, whereas Miyoshi myopathy involves the posterior compartment muscles of the lower limb [4,5]. The progressive muscle degeneration caused by this disease results in mobility impairment and disability that increases in severity during advanced stages. However, most have an approximately normal life span. The age of onset, rate of progression, and severity of this disease are highly variable and unpredictable in patients with dysferlinopathy, indicating a role for environmental and epigenetic factors. Although the exact prevalence of dysferlinopathy is not known, most LGMDs are rare with an estimated prevalence ranging from 0.07-0.43 per 100,000 people [6]. Currently, there is no cure for this disease, and existing treatment strategies are aimed at managing complications and prolonging life span.

In recent years, scientists have discovered a group of heterogeneous RNA molecules called noncoding RNAs (ncRNAs) that participates in many physiological functions and disease processes. Interestingly, only 2% of the eukaryotic genome is transcribed to functional protein and the remaining 98% is considered as nonprotein coding RNAs or ncRNAs [7]. NcRNAs are broadly classified into (1) structural ncRNAs that include ribosomal RNAs, transfer RNAs, small nuclear RNAs, and small nucleolar RNAs and (2) regulatory ncRNAs that include small ncRNAs (shorter than 200 nucleotides) and long noncoding RNAs (lncRNAs). LncRNAs are transcripts longer than 200 nucleotides and participate in regulating gene expression through a variety of mechanisms [8]. For example, they participate in chromatin remodeling through the recruitment of polycomb repressive complex 2, causing gene repression [9,10]. In addition, they regulate the binding of transcription factors to the DNA loci by forming hybridization structures with the DNA. Some of the lncRNAs are known to function as competing endogenous RNAs and sponge-specific microRNAs to regulate gene expression [11-13]. Interestingly, they can reduce the stability of messenger RNAs (mRNAs) or increase their translation through different mechanisms depending on their subcellular localization, interacting partners, and local environments in the cells [14]. Some recent studies have described the function and mechanism of selected lncRNAs in the pathogenesis of specific diseases, including cardiac hypertrophy [15], osteoarthritis [16], and fascioscapulohumeral muscular dystrophy [17]. Further, some studies have revealed a few muscle-specific lncRNAs that are involved in regulating muscle cell growth and differentiation [13,18]. Nevertheless, their expression signature, function, and contribution to the disease process of dysferlinopathy are not well studied.

This paper presents the results of the analysis on the lncRNAs expression profile between wild-type and dysferlin-deficient murine myoblasts using a microarray. To confirm our microarray results, we validated some of the lncRNAs that were differentially expressed with the help of quantitative real-time polymerase chain reaction (qRT-PCR). Additionally, using bioinformatics, this paper also annotates the possible cellular function and interactions for these lncRNAs.

## Methods

### Cell Culture

The C2C12 cell line was a generous gift from Dr Robert H. Brown, Department of Neurology, University of Massachusetts [19]. A fixed density of wild-type and dysferlin-deficient C2C12 cells were cultured in T75 flasks in growth media containing DMEM supplemented with 20% bovine growth serum (HyClone) and 1% penicillin-streptomycin. For the dysferlin-deficient cells, 1.5 µg/mL puromycin was added to the growth media. Media were changed every 2 days and cells were split before they reach confluence in order to avoid differentiation and cell death.

### RNA Extraction

Total RNA from the wild-type and dysferlin-deficient C2C12 cells was extracted using the RNeasy plus kit (Qiagen) following the manufacturer’s instructions. A NanoDrop ND-1000 spectrophotometer was used to measure the quality and quantify the RNA samples. The integrity of the RNA samples was assessed by agarose gel electrophoresis.

### Microarray

LncRNA and mRNA expression profiling were performed using Arraystar Mouse LncRNA Microarray V3.0 containing 21,486 lncRNA and 18,921 mRNA probes. The lncRNA probes were created based on the information derived from reputable transcriptome public databases, such as Refseq, UCSC known genes, and Ensembl and landmark publications. The percentage of probes made for each category of lncRNAs is given in Table 1. For accurate identification of individual transcripts, probes that will bind to specific exons or splice junctions were designed. For the purpose of hybridization quality control, the array also contained positive probes (for housekeeping genes) and negative probes.

**Table 1:** Percentage of lncRNA probes designed for each category.

LncRNA category	Probes designed, n (%)
Bidirectional	993 (4.6)
Exon sense-overlapping	7451 (34.7)
Intergenic	9183 (42.7)
Intron sense-overlapping	497 (2.3)
Intronic antisense	1425 (6.6)
Natural antisense	1937 (9)
Total	21,486 (100)

### RNA Labeling and Array Hybridization

Sample labeling and array hybridization were performed according to the Agilent One-Color Microarray-Based Gene Expression Analysis protocol (Agilent Technology) with minor modifications. Briefly, ribosomal RNA was first removed from the total RNA sample, and then mRNA was purified using the mRNA-ONLY Eukaryotic mRNA Isolation Kit (Epicentre Biotechnologies). The purified samples were then amplified and transcribed into fluorescent complementary RNA (cRNA) along the entire length of the transcripts without 3’ bias using a mixture of oligo(dT) and random priming method (Arraystar Flash RNA Labeling Kit). This was followed by cRNA purification using the RNeasy Mini Kit (Qiagen) following the manufacturer’s instructions. A NanoDrop ND-1000 spectrophotometer was used to measure the concentration and specific activity of the labeled cRNAs (pmol Cy3/μg cRNA). 5 μL of 10 × Blocking Agent and 1 μL of 25 × Fragmentation Buffer were added to 1 μg of the labeled cRNA to fragment, and then the mixture was heated at 60 °C for 30 minutes. To achieve the desired dilution, 25 μL of 2 × GE Hybridization buffer was added. 50 μL of hybridization solution was used to assemble the probes on the microarray slides. The slides were incubated for 17 hours at 65 °C in an Agilent Hybridization Oven. The hybridized arrays were washed, fixed, and scanned using the Agilent DNA Microarray Scanner (part number G2505C).

### Data Analysis

The array images were acquired and analyzed using the Agilent Feature Extraction software (version 11.0.1.1). GeneSpring GX software package (version 12.1; Agilent Technologies) was used for further data processing and quantile normalization. Further analysis was done on samples that had flags in Present or Marginal (“All Targets Value”) values. Differentially expressed lncRNAs and mRNAs were identified through fold change (FC) filtering between the samples. The cutoff values were |FC|≥2, where FC values in linear scale (not in log_2_ scale) were calculated based on the normalized intensities.

### GO Analysis and Pathway Analysis

GO analysis was derived from Gene Ontology [20], which has 3 structured networks of defined terms describing gene product attributes. The *P* value denotes the significance of GO term enrichment in the differentially expressed mRNA list. Pathway analysis for differentially expressed mRNAs was based on the latest KEGG (Kyoto Encyclopedia of Genes and Genomes) [21] database. For both the GO and pathway analysis, a *P* value of <.05 was considered to be statistically significant.

### qRT-PCR Analyses

Total RNA was extracted from the wild-type and dysferlin-deficient myoblast cells using RNeasy Mini Kit (Qiagen) following the manufacturer’s instructions. They were then reverse transcribed using a reverse transcription kit (QuantaBio), and qRT-PCR analyses was completed using SYBR Green FastMix (QuantaBio) and StepOnePlus RT-PCR instrument. The sequences of the primers used in this study are given in Table 2. GAPDH was used for normalization, and the FC was calculated using the 2^-ΔΔCt^ method. The results presented are an average from 3 biological replicates.

**Table 2:** List of primers used for quantitative real-time polymerase chain reaction.

Long noncoding RNA	Primer sequence (5’ to 3’)
TnnT3	Fwd - AGCTCCAAGCCCTCATTGAC Rev - CTCCTCCTCTCTTCTGGCCT
H19	Fwd - ATCCTGGAGCCAAGCCTCTA Rev - TCACGGGTGCTTTGAGTCTC
XLOC_011052	Fwd - CCAGGAAGTTGAAGCAGGAG Rev - CGGAGAACAATGTGGTGGTA
XLOC_008220	Fwd - TTTCACTTGCTGGCTTTTGA Rev - ACTCCCAGCCAGCTGTGTC
AK085239	Fwd - CCATCCCTACACTGCAGCAA Rev - GTTGGGAAAGCATGGCTGTG
AK032137	Fwd - CCTTGGAGTGAAGTGGCCAT Rev - CTCTCTCCTCCCTTGCCTCT
Trak2	Fwd - CCTAGCTCCGGTTTCCCATC Rev - CGGTTTGTGATGGATTCGCC

### Network Analysis

MetaCore software (2021 version; GeneGo Inc) was used for network analysis. Based on the FCs between the knockout (KO) versus wild-type lncRNAs, the top 25 upregulated and top 25 downregulated lncRNAs were selected from the data set (Multimedia Appendix 1 shows the gene symbols [lncRNA identifiers] and FCs). The selected data were uploaded to the software’s build network tab under the selection *Build Network for Single Gene/Protein/Compound or a List*. Biological networks were built using the Analyze Network Algorithm with default MetaCore settings. The key parameters used for generating the output are the relative enrichment obtained from the uploaded data and the relative saturation of the networks with canonical pathways.

## Results

### Differentially Expressed LncRNAs

**Figure 1:**
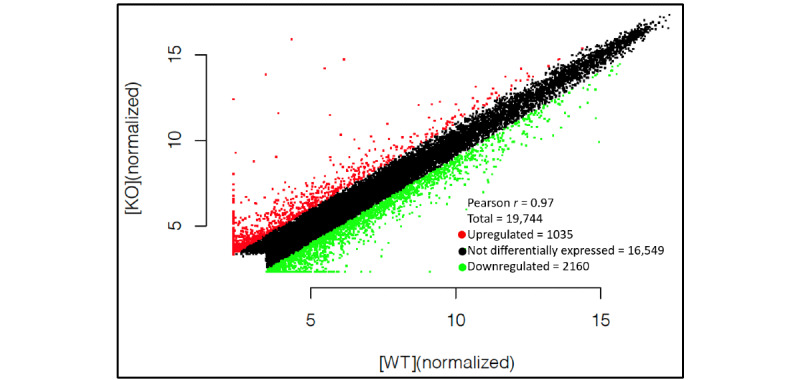
Differentially expressed lncRNAs based on microarray data. Scatter plot of differentially expressed lncRNAs in dysferlin-deficient murine myoblasts compared to normal controls. Red points represent upregulated lncRNAs and green points represent downregulated lncRNAs in dysferlin-deficiency myoblasts with a fold change greater than 2.0. KO: knockout; LncRNA: long noncoding RNA; WT: wild type.

**Figure 2:**
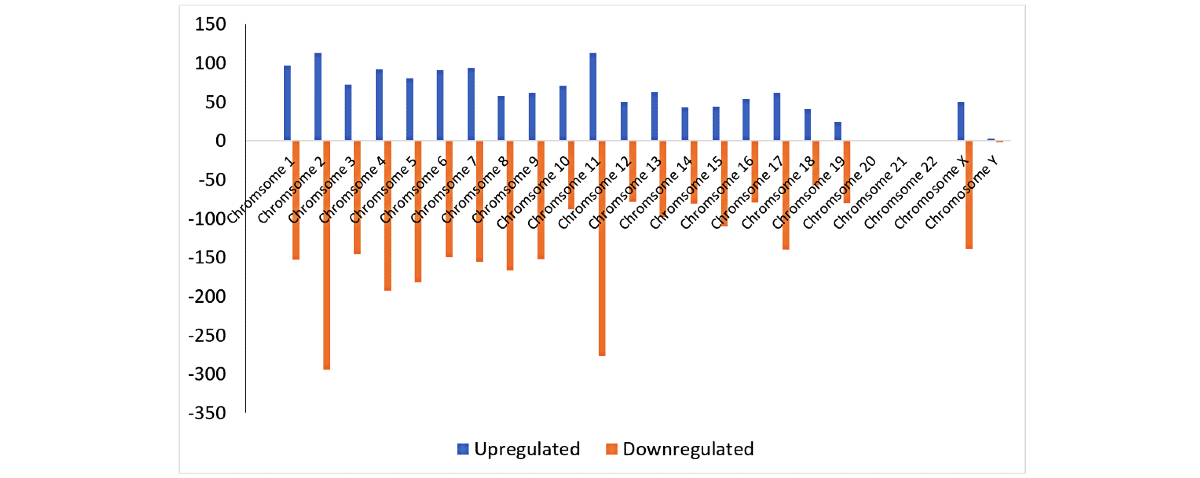
Chromosomal distribution of differentially expressed lncRNAs in dysferlin-deficient myoblasts, showing upregulated (blue) and downregulated (orange) lncRNAs in each chromosome. lncRNA: long noncoding RNA.

The microarray revealed that of the 19,744 lncRNAs tested, 3195 were differentially expressed in the dysferlin-deficient cells compared to the wild-type cells. Among these lncRNAs, 1035 were upregulated and 2160 were downregulated (Figure 1). Interestingly, 59 lncRNAs were found to have an FC of greater than 10. The most upregulated lncRNA was humanlincRNA0955 (FC=3077.01), and the most downregulated was AK085239 (FC=110.64). Analyzing the chromosomal distribution of the differentially expressed lncRNAs revealed that they are mostly unequally distributed (Figure 2). There were 2 lncRNAs assigned to the mitochondrial genome. Chromosome 2 contained the largest number of dysregulated lncRNAs (295/3195, 9.2%) and included 80 upregulated and 215 downregulated lncRNAs in the dysferlin-deficient cells compared to wild-type cells.

### LncRNA Classification

LncRNAs are classified based on their relative position to the nearby protein-coding genes. In this study, we identified differentially expressed lncRNAs that were distributed among 4 different categories: sense, intergenic, antisense, and bidirectional. The sense and antisense lncRNAs are transcribed from the sense and antisense strands of the DNA, respectively. Intergenic lncRNAs are derived from DNA sequences between genes, and bidirectional lncRNAs use the same promoter as the protein-coding genes but are transcribed in the opposite direction [22]. The majority of the differentially expressed lncRNAs were sense (total: 1385/3195, 43.3%; upregulated: 272/1035; downregulated: 1113/2160) and intergenic (total: 1224/3195, 38.3%; upregulated: 513/1035; and downregulated: 711/2160) lncRNAs.

### Differentially Expressed mRNAs

**Figure 3:**
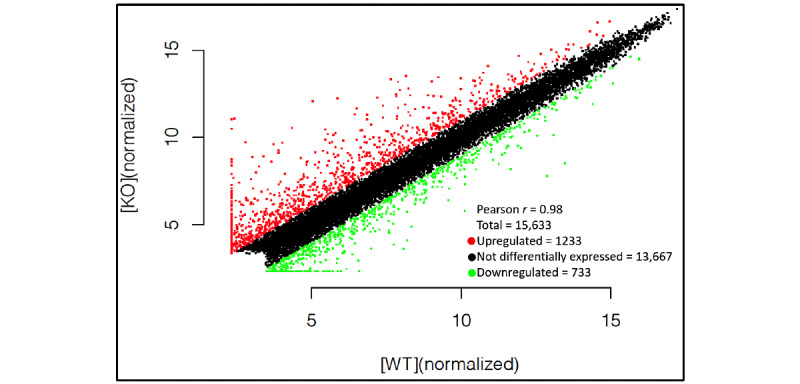
Differentially expressed mRNAs based on microarray data. Scatter plot of differentially expressed mRNAs in dysferlin-deficient murine myoblasts compared to normal controls. Red points represent upregulated mRNAs and green points represent downregulated lncRNAs in dysferlin-deficiency myoblasts with a fold change greater than 2.0. KO: knockout; mRNA: messenger RNA; WT: wild type.

**Figure 4:**
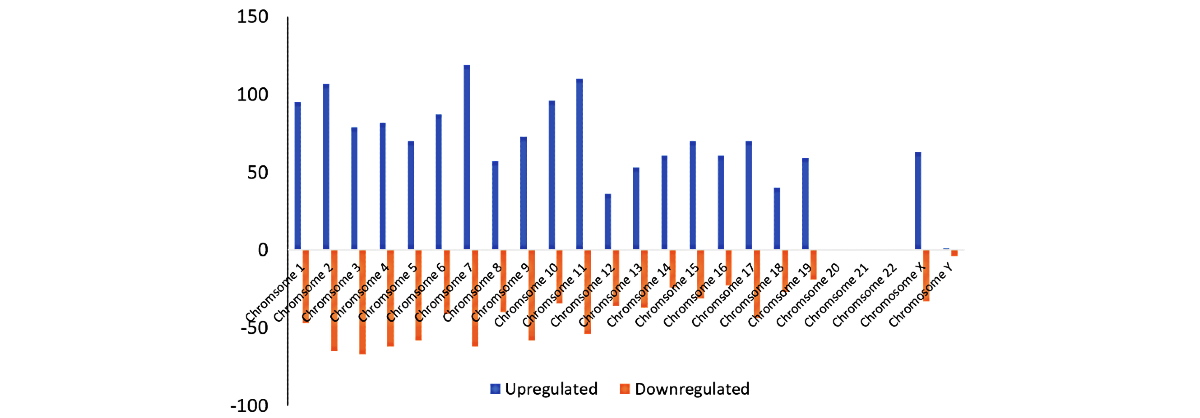
Chromosomal distribution of differentially expressed mRNAs in dysferlin-deficient myoblasts, showing upregulated (blue) and downregulated (orange) mRNAs in each chromosome. mRNA: messenger RNA.

Of the 15,633 mRNAs screened by the microarray, 1966 were differentially expressed in the dysferlin-deficient cells compared to the wild-type cells. Among the 1966 mRNAs, 1233 were upregulated and 733 were downregulated (Figure 3). A total of 126 mRNAs had an FC greater than 10. The most upregulated mRNA was Gm11565 (FC=422.77), and the most downregulated mRNA was Lce1h (FC=33.92). Around 9.2% (181/1966; 119 upregulated and 62 downregulated) of the probed mRNAs were found on chromosome 7, followed by 8.7% (171/1966) on chromosome 2 (Figure 4).

### qRT-PCR Validation of Microarray Results

**Figure 5:**
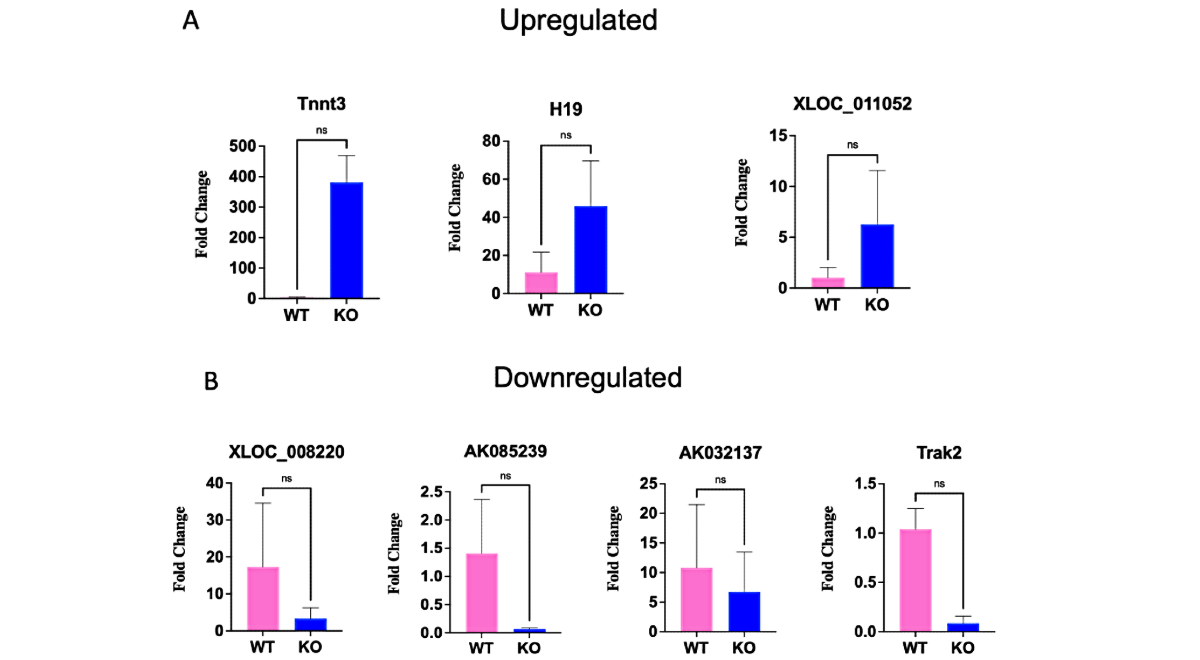
Quantitative real-time polymerase chain reaction (qRT-PCR) validation of microarray results. The bar graphs show the fold changes in the knockout samples compared to the wild type. Error bars represent SD (N=3). KO: knockout; WT: wild type.

To confirm the reliability of the microarray results, we randomly selected 6 differentially expressed lncRNAs—3 upregulated (TnnT3, H19, and XLOC_011052) and 4 downregulated (XLOC_008220, AK085239, AK032137, and Trak2)—from the microarray results. The analyses’ results were consistent with the direction of change of the microarray results (Figure 5).

### GO and KEGG Pathway Analysis of the Differentially Expressed mRNAs

**Figure 6:**
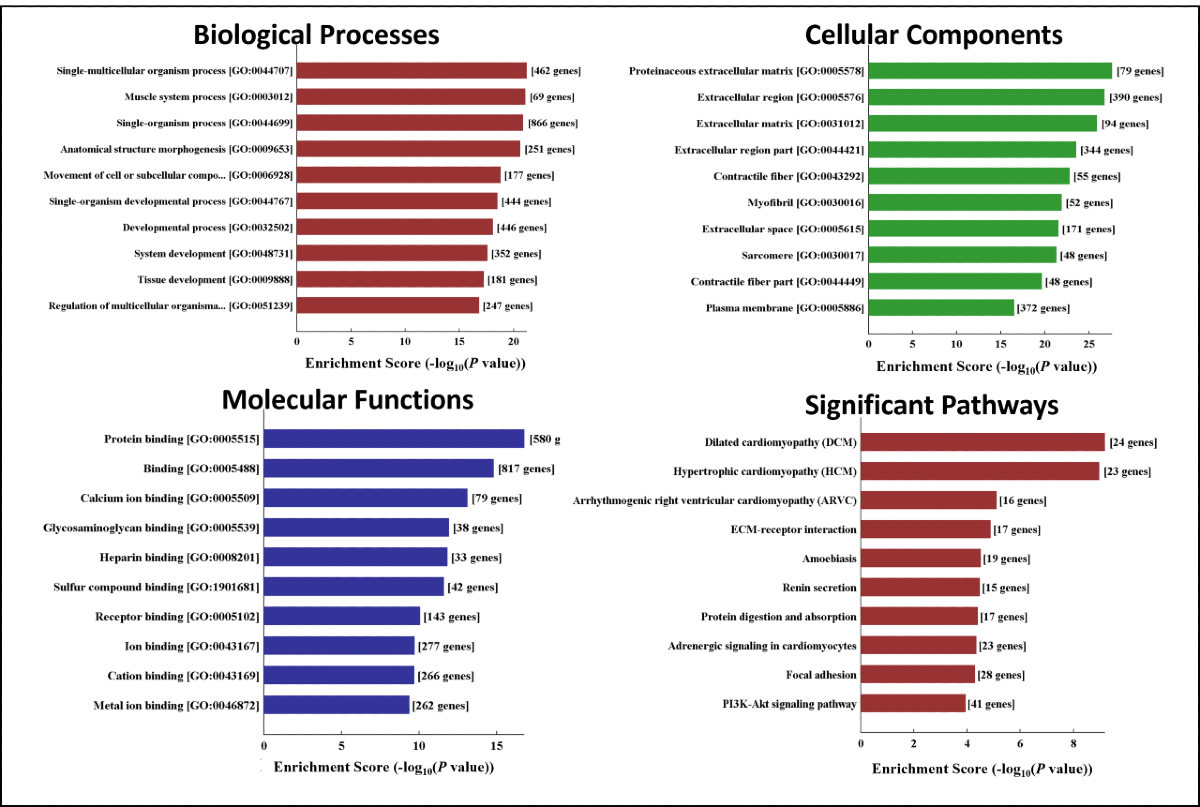
Gene Ontology (GO) analysis and Kyoto Encyclopedia of Genes and Genomes (KEGG) analysis of upregulated genes. mRNA: messenger RNA.

**Figure 7:**
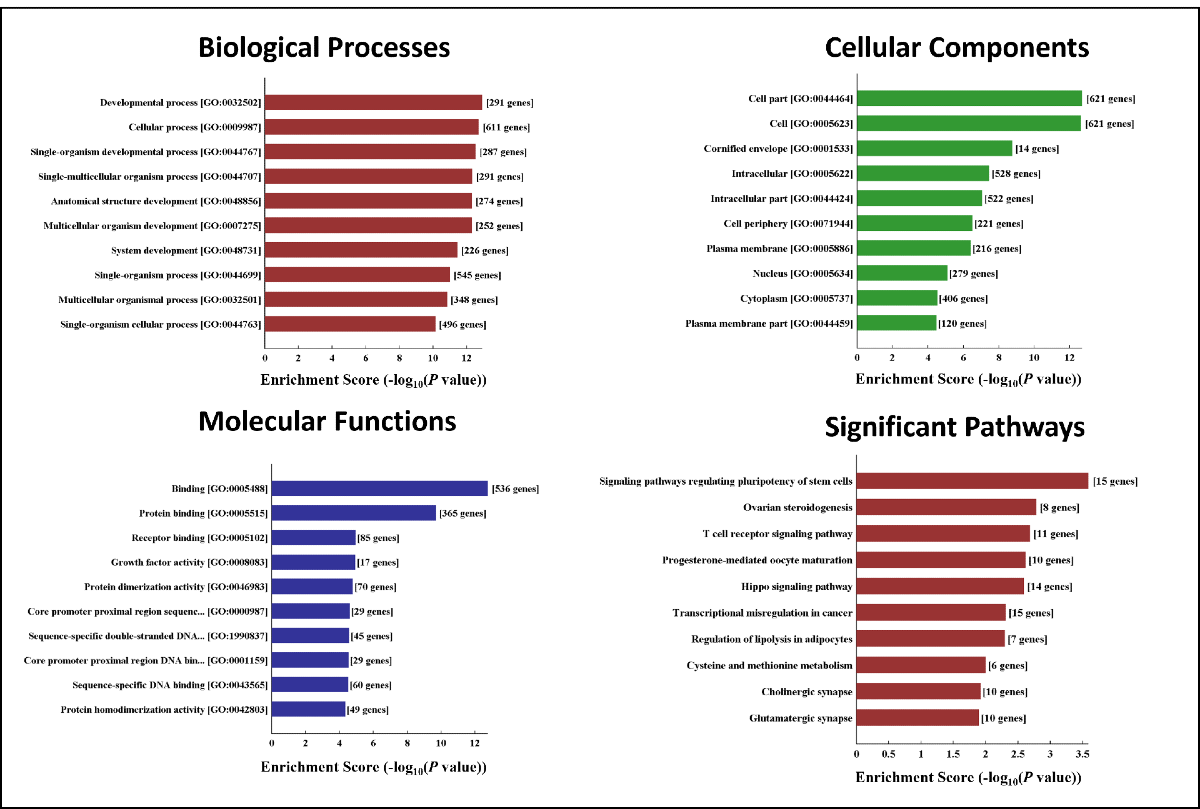
Gene Ontology (GO) analysis and Kyoto Encyclopedia of Genes and Genomes (KEGG) analysis of downregulated genes. mRNA: messenger RNA.

GO analysis was performed to understand the functional role of the differentially expressed mRNAs (Figures 6-7). The analysis covered 3 domains: biological process, cellular component, and molecular function. The most significantly enriched terms in this study were *protein binding* and *binding*. Additionally, *proteinaceous extracellular matrix*, *extracellular region*, and *cell part* were enriched at the cellular component level, and *single-multicellular organism process*, *muscle system process*, and *developmental process* were significantly enriched at the biological process level. We used enrichment scores to rank the pathways involved in dysferlin deficiency. The top 10 enriched pathways associated with the upregulated and downregulated genes are shown in Figures 6 and 7, respectively.

### Network Analysis

**Figure 8:**
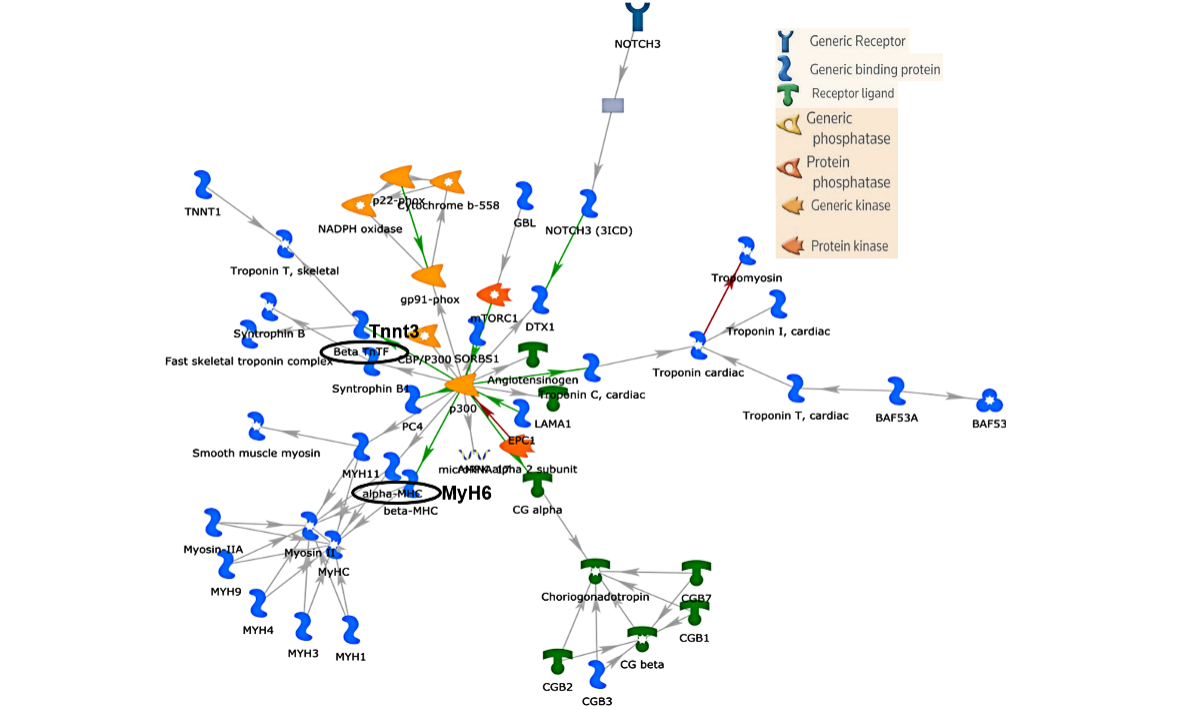
The top scored (by the number of pathways) network obtained from the top 13 upregulated long noncoding RNAs (lncRNAs). Green arrows indicate activation effect, red arrows indicate inhibition effect, and gray arrows indicate unspecified effects. The lncRNAs beta TnTF (Tnnt3) and alpha-MHC (MyH6) are circled in black.

**Figure 9:**
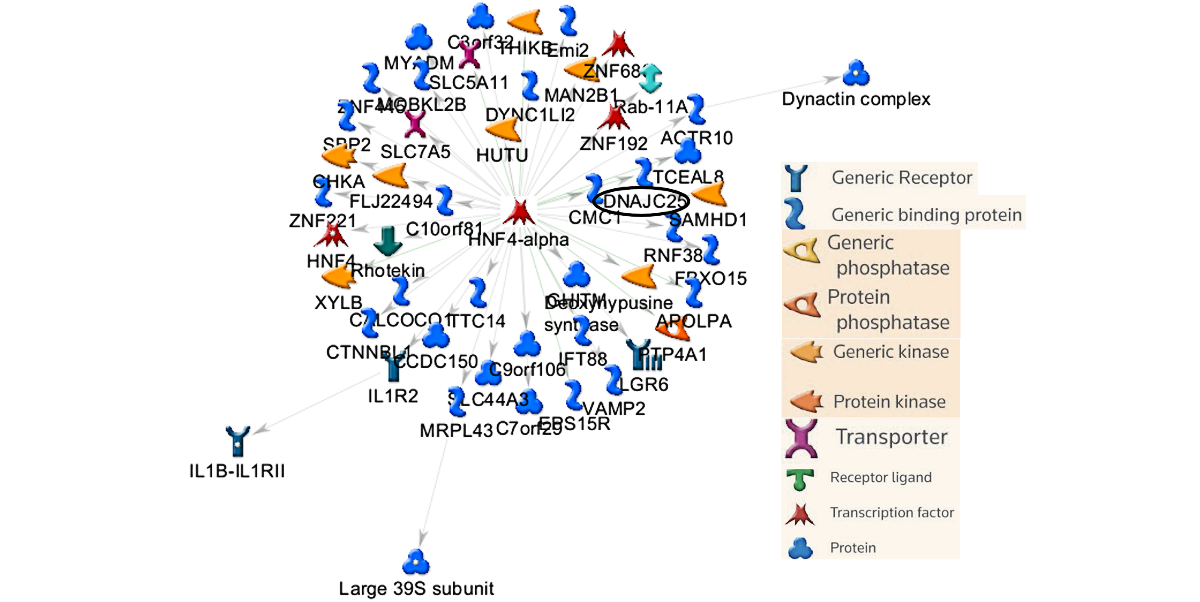
The top scored (by the number of pathways) network from the top 16 downregulated long noncoding RNAs (lncRNAs). Gray arrows indicate unspecified effects. The lncRNA DNAJC25 is circled in black.

Network analysis on lncRNAs was performed to evaluate their associations with transcription factors, receptors, protein complex, small molecules, and biochemical pathways. The top 25 upregulated lncRNAs (humanlincRNA0955, Trak2, 4930480G23Rik, AK078726, AK037210, AK085419, mouselincRNA0086, Tnnt3, AK038305, Filip1, Gm20485, Fam189a1, AK135257, Myh6, Coro2b, H19, AK045129, Enpep, AK144424, AK143389, Gm5401, AK035065, AK009210, humanlincRNA1720, and AK034241) were selected for network analysis. Of these 25 lncRNAs, 13 lncRNAs (Trak2, 4930480G23Rik, AK085419, Tnnt3, Filip1, Fam189a1, AK135257, Myh6, Coro2b, H19, Enpep, AK009210, and AK034241) were recognized by MetaCore, and these specific lncRNAs were used for network analysis. A total of 8 statistically significant network objects were identified from upregulated lncRNAs (Table 3). The top scored network consisted of input lncRNAs Tnnt3, listed as Beta TnTF (Tnnt3), and MyH6, listed as alpha MHC. The top network also included Troponin cardiac (Tnnt2), Troponin T (Tnnt2), and p300 (Table 3 and Figure 8). Interestingly, the top upregulated network was associated 56.2% with muscle system process and 50% with muscle contraction functions. The top 25 downregulated lncRNAs (AK085239, AK135501, AK032137, mouselincRNA1640, AK005833, XLOC_011793, 5830416P10Rik, Gm15389, AK078320, AK017917, Prl2c5, AK144783, AK155441, AK076675, Vmn2r-ps67, Dnajc2, Atrn, Vwc2l, AK032666, DQ687127, humanlincRNA2050, Gm14879, AK019774, Reps2, and AK041109) were selected for network analysis. Of these 25 lncRNAs, 16 lncRNAs (AK085239, AK135501, AK032137, AK005833, AK078320, AK017917, Prl2c5, AK076675, Vmn2r-ps67, Dnajc2, Atrn, Vwc2l, AK032666, AK019774, Reps2, and AK041109) were recognized by MetaCore, and these specific lncRNAs were used for network analysis. A total of 3 statistically significant network objects were identified from downregulated lncRNAs (Table 4 and Figure 9). The top scored network consisted of input lncRNA Dnajc2, listed as Dnajc25. The top network also included LGR6, HNF4-alpha, MRPL43, and Emi2.

**Table 3:** Statistically significant networks obtained from 13 upregulated long noncoding RNAs. The sequence of network objects is prioritized based on the number of fragments of canonical pathways on the network.

No., name, Gene Ontology processes (%; *P* value)			Total nodes		Seed nodes		*P* value
**1. Troponin T, cardiac, Beta TnTF, Troponin cardiac, alpha-MHC, p300**			50		8		7.97 × 10^-26^
	muscle filament sliding (35.4; 9.832 × 10^-35^)						
	actin-myosin filament sliding (35.4; 1.459 × 10^-34^)						
	muscle system process (56.2; 1.447 × 10^-33^)						
	muscle contraction (50; 6.408 × 10^-31^)						
	actin-mediated cell contraction (35.4; 9.680 × 10^-27^)						
**2. H19, FILIP, AKT1, Tip60, miR-29a-3p**			50		2		4.52 × 10^-06^
	histone H4 acetylation (25; 1.131 × 10^-16^)						
	peptidyl–amino acid modification (50; 1.613 × 10^-16^)						
	histone acetylation (25; 1.170 × 10^-13^)						
	internal peptidyl-lysine acetylation (25; 2.149 × 10^-13^)						
	peptidyl-lysine acetylation (25; 2.712 × 10^-13^)						
**3. NckAP1, c-Myc, PCNT1, CD133, SHB**			50		2		5.62 × 10^-06^
	viral transcription (34; 5.579 × 10^-27^)						
	viral gene expression (34; 7.051 × 10^-26^)						
	peptidyl-lysine modification (34; 2.567 × 10^-18^)						
	viral process (46; 1.024 × 10-17)						
	biological process involved in symbiotic interaction (46; 1.493 × 10^-16^)						
**4. c-Myc, POM121, NUP54, FZD9, NUP37**			50		1		3.32 × 10^-03^
	viral transcription (40.4; 8.401 × 10^-32^)						
	viral gene expression (40.4; 1.476 × 10^-30^)						
	intracellular transport of virus (31.9; 1.691 × 10^-27^)						
	transport of virus (31.9; 1.250 × 10^-26^)						
	multiorganism localization (31.9; 2.301 × 10^-26^)						
**5. H19, Cyclin D1, ITGB4, LKB1, GLUT4**			50		1		3.39 × 10^-03^
	response to organic cyclic compound (61.4; 6.074 × 10^-19^)						
	tissue development (68.2; 1.553 × 10^-18^)						
	response to abiotic stimulus (61.4; 3.091 × 10^-18^)						
	gland development (45.5; 6.551 × 10^-18^)						
	positive regulation of cellular metabolic process (79.5; 1.774 × 10^-17^)						
**6. WRC, c-Myc, OTX2, APEX, Folliculin**			50		1		3.46 × 10^-03^
	positive regulation of nitrogen compound metabolic process (77.1; 1.738 × 10^-18^)						
	positive regulation of macromolecule metabolic process (79.2; 5.340 × 10^-18^)						
	positive regulation of cellular process (91.7; 1.152 × 10^-17^)						
	positive regulation of biological process (93.8; 2.262 × 10^-17^)						
	positive regulation of protein metabolic process (60.4; 2.337 × 10^-17^)						
**7. Y549 (GRIF1), PPARGC1 (PGC1-alpha), OGT (GlcNAc transferase), MMP-9, mTOR**			50		1		3.54 × 10^-03^
	positive regulation of macromolecule metabolic process (87; 7.785 × 10^-22^)						
	positive regulation of DNA-templated transcription (67.4; 1.717 × 10^-21^)						
	positive regulation of nucleic acid–templated transcription (67.4; 4.954 × 10^-21^)						
	positive regulation of RNA biosynthetic process (67.4; 5.018 × 10^-21^)						
	negative regulation of nitrogen compound metabolic process (76.1; 6.518 × 10^-21^)						
**8. Y549 (GRIF1), c-Myc, mRNA intracellular, NUP35, RAE1**			50		1		3.54 × 10^-03^
	viral transcription (44.9; 5.321 × 10^-38^)						
	viral gene expression (44.9; 1.541 × 10^-36^)						
	Signal Recognition Protein (SRP)–dependent cotranslational protein targeting to membrane (28.6; 2.873 × 10^-22^)						
	cotranslational protein targeting to membrane (28.6; 4.992 × 10^-22^)						
	viral process (53.1; 6.767 × 10^-22^)						

**Table 4:** Statistically significant networks obtained from 16 downregulated long noncoding RNAs. The sequence of network objects is prioritized based on the number of fragments of canonical pathways on the network.

No., name, Gene Ontology processes (%; *P* value)			Total nodes		Seed nodes		*P* value
**1. DNAJC25, LGR6, HNF4-alpha, MRPL43, Emi2**			50		2		1.98 × 10^-06^
	negative regulation of activation of Janus kinase activity (4.7; 9.716 × 10^-06^)						
	retrograde axonal transport of mitochondrion (4.7; 1.941 × 10^-05^)						
	negative regulation of interleukin-1 alpha production (4.7; 3.231 × 10^-05^)						
	purine deoxyribonucleotide catabolic process (4.7; 4.841 × 10^-05^)						
	negative regulation of interleukin-1-mediated signaling pathway (4.7; 6.770 × 10^-05^)						
**2. DNAJC27, GATA-4, ERK1/2, Decanoyl-CoA + Acetyl-CoA = 3-Oxo-dodecanoyl-CoA + CoA, BAF250A**			50		1		1.84 × 10^-03^
	cellular response to organic cyclic compound (50; 4.858 × 10^-18^)						
	cellular response to lipid (47.6; 7.417 × 10^-17^)						
	response to lipid (57.1; 8.792 × 10^-17^)						
	cardiocyte differentiation (31; 1.763 × 10^-16^)						
	response to organic cyclic compound (57.1; 5.534 × 10^-16^)						
**3. MPP11, NANOG, HOXB1, PECAM1, VISA**			50		1		2.09 × 10^-03^
	muscle system process (34; 6.752 × 10^-17^)						
	platelet aggregation (18; 1.312 × 10^-13^)						
	homotypic cell-cell adhesion (18; 1.131 × 10^-12^)						
	regulation of muscle system process (26; 2.361 × 10^-12^)						
	muscle hypertrophy (16; 4.725 × 10^-12^)						

## Discussion

Abnormal expression of specific lncRNAs have been described in various diseases including certain types of muscular dystrophies, such as Duchenne, myotonic, and facioscapulohumeral muscular dystrophies [17,23,24]. However, to date, there is no information on the lncRNAs associated with dysferlinopathy or any type of LGMD. This study is the first to screen and report the difference in the expression patterns of lncRNAs and mRNAs in wild-type and dysferlin-deficient myoblasts. Thereafter, we analyzed the microarray results and performed bioinformatics analysis to understand their possible interactions and functions.

Our results show that there were a high number of lncRNAs and mRNAs that were differentially expressed due to dysferlin deficiency. There were more downregulated than upregulated lncRNAs, but more upregulated than downregulated mRNAs. We validated the microarray results by testing the expression of several lncRNAs, which were consistent for the direction of change, although there were small inconsistencies in the magnitude of FCs. This is expected due to the differences between the 2 methods, the different normalization strategies used, and their inherent pitfalls.

The chromosomal distribution of the differentially expressed lncRNAs and mRNAs were not equal. Chromosomes 2 and 11 had a greater percentage of lncRNAs, whereas chromosomes 2 and 7 had a greater percentage of mRNAs that were differentially expressed. It is interesting to note that the dysferlin gene is located in chromosome 2 in humans and in chromosome 6 in mice. It is tempting to posit that at least some of the differentially expressed lncRNAs and mRNA gene products may directly regulate the expression or function of the dysferlin protein.

According to their position and directionality of transcription in relation to other genes, lncRNAs can be classified into multiple subgroups such as sense lncRNAs, antisense lncRNAs, bidirectional lncRNAs, and long-intergenic noncoding RNAs (lincRNAs). Interestingly, a majority of the differentially expressed lncRNAs identified in this study are sense lncRNAs (43.3%) or intergenic lncRNAs (38.3%). Together, they contribute to more than three-quarters of the total lncRNAs that were differentially expressed. LincRNAs are ncRNAs that are transcribed from regions nearby the protein-coding genes without overlapping them. They are known to be highly tissue-specific and can regulate the nearby protein-coding genes and genes far away from them [25]. Sense lncRNAs are those that are transcribed from the sense strand of DNA, and they may overlap or contain an entire protein-coding gene sequence within them. The differential expression of these 2 types of lncRNAs suggests that these lncRNAs may be involved in regulating the protein-coding genes that are involved in the progression of dysferlinopathy. Since the lincRNAs are more tissue-specific, future studies may focus on evaluating any correlation between their expression and tissue involvement or disease severity in dysferlinopathy.

As lncRNAs are regulatory molecules, the differentially expressed lincRNAs and sense lncRNAs could possibly control the expression of the nearby or overlapping genes through multiple mechanisms. Hence, it is tempting to predict that the function of many of the novel lncRNAs identified in this study could be related to the function of the associated genes. From our GO and pathway analysis, some of the differentially expressed mRNAs were functionally related to skeletal muscle contraction (16 genes), skeletal muscle relaxation (12 genes), regulation of muscle contraction (5 genes), and actin-myosin filament sliding (5 genes). The related signaling pathways included the PI3K-Akt signaling pathway (41 genes), Hippo signaling pathway (14 genes), and pathways that control the pluripotency of stem cells (15 genes).

This study has several limitations. The dysferlin-deficient murine myoblasts used in this study had dysferlin silenced using a short hairpin RNA (shRNA). Hence, these cells could have low-level dysferlin expression that may have influenced the differential expression. Since there is very minimal prior research in this area, the significance and interactions of the differentially expressed lncRNAs are only predicted and not confirmed. Additionally, the microarray was performed using pooled RNA from 3 biological replicates, and therefore, the statistical significance for the FCs could not be calculated. To note, puromycin is known to cause changes in gene expression [26,27] and cellular stress [28,29] in mammalian cell lines. Hence, its addition to the KO cells may have contributed to the differential lncRNA and mRNA expression in the myoblasts tested. Although the shRNA construct that was used to establish the KO cell line carried the puromycin acetyltransferase (pac) gene and is expected to confer resistance to puromycin, the efficiency can vary depending on the cell lines [30]. Finally, since the study has been conducted using murine myoblasts, the differentially expressed lncRNA signature may differ in human-derived, dysferlin-deficient myoblasts.

In conclusion, this is the first report illustrating the lncRNA signature in dysferlinopathy. Our results highlight that lncRNAs are involved in regulating the gene expression and critical biological functions such as muscle contraction and relaxation in dysferlinopathy. Future studies focusing on deciphering the exact mechanisms that contribute to the regulatory function of these lncRNAs will be interesting and add more to our understanding of this incurable disease.

## Appendix

Multimedia Appendix 1 Gene symbols and fold changes.

## Abbreviations

cRNA: complementary RNA
FC: fold change
GO: Gene Ontology
KEGG: Kyoto Encyclopedia of Genes and Genomes
KO: knockout
LGMD: limb-girdle muscular dystrophy
lincRNA: long-intergenic noncoding RNA
lncRNA: long noncoding RNA
mRNA: messenger RNAs
ncRNA: noncoding RNAs
qRT-PCR: quantitative real-time polymerase chain reaction
