# Supplementary material for: Differential Expression of Long Noncoding RNAs in Murine Myoblasts After Short Hairpin RNA-Mediated Dysferlin Silencing In Vitro: Microarray Profiling
Source: JMIR Bioinform Biotechnol. 2022 Jun 17;3(1):e33186. doi: 10.2196/33186 (PMC11135227; doi:10.2196/33186)
Supplement: Multimedia Appendix 1 [file bioinform_v3i1e33186_app1.pdf]

Multimedia Appendix 1. Gene symbols and fold changes.

GeneSymbol	source	RNAlength
humanlincRNA0955	lincRNA	15316
Trak2	Ensembl	1018
4930480G23Rik	RefSeq	1259
AK078726	Genbank	2175
AK037210	Genbank	1994
AK085419	Genbank	1120
mouselincRNA0086	lincRNA	5325
Tnnt3	Ensembl	1011
AK038305	Ensembl	1011
Filip1	Ensembl	1011
Gm20485	Ensembl	1011
Fam189a1	Ensembl	1011
AK135257	Ensembl	1011
Myh6	Ensembl	1011
Coro2b	Ensembl	1011
H19	Genbank	3598
AK045129	Ensembl	3775
Enpep	Ensembl	394
AK144424	Ensembl	3842
AK143389	Genbank	2121
XLOC_005827	UCSC_knowngene	168
Gm5401	Ensembl	1872
AK035065	Ensembl	1853
AK009210	Ensembl	935
humanlincRNA1720	Genbank	2880
AK034241	Ensembl	2655

Regulation	Fold Change (KO vs Wt)
up	3077.0
up	1354.1
up	1080.8
up	426.7
up	384.3
up	209.0
up	116.4
up	57.1
up	57.1
up	57.1
up	57.1
up	57.1
up	57.1
up	57.1
up	57.1
up	53.7
up	52.6
up	51.9
up	37.6
up	34.5
up	27.3
up	21.5
up	20.6
up	19.5
up	19.0
up	17.5

GeneSymbol	source	RNAlength	Regulation
AK085239	Genbank	2459	down
AK135501	Genbank	1572	down
AK032137	Genbank	1756	down
mouselincRNA1640	lincRNA	11824	down
AK005833	UCSC_knowngene	457	down
XLOC_011793	Alexander et al 2013	2269	down
5830416P10Rik	RefSeq	5292	down
Gm15389	Ensembl	666	down
AK078320	Genbank	917	down
AK017917	Genbank	1384	down
Prl2c5	Ensembl	485	down
AK144783	Genbank	2788	down
AK155441	Genbank	1685	down
AK076675	UCSC_knowngene	1337	down
Vmn2r-ps67	Ensembl	385	down
Dnajc2	Ensembl	3758	down
Atrn	Ensembl	4139	down
Gas5	Ensembl	872	down
Vwc2l	Ensembl	4191	down
AK032666	Genbank	2233	down
DQ687127	UCSC_knowngene	83	down
humanlincRNA2050	lincRNA	458	down
Gm14879	Ensembl	507	down
AK019774	Ensembl	530	down
Reps2	UCSC_knowngene	397	down
AK041109	Ensembl	667	down

Fold Change (KO vs Wt)
110.6
103.4
33.0
26.5
21.4
21.0
18.5
15.8
14.5
14.4
12.6
12.5
12.3
11.9
11.8
11.8
11.7
11.6
11.3
11.0
10.7
10.6
10.5
10.2
10.2
9.9
